# Establishment and identification of an animal model of Hirschsprung disease in suckling mice

**DOI:** 10.1038/s41390-023-02728-6

**Published:** 2023-07-17

**Authors:** Chaoting Lan, Yuxin Wu, Yanqing Liu, Ning Wang, Meiling Su, Dingjiang Qin, Weiyong Zhong, Xinying Zhao, Yun Zhu, Qiuming He, Huimin Xia, Yan Zhang

**Affiliations:** 1 Department of Pediatric Surgery, Guangdong Provincial Key Laboratory of Research in Structural Birth Defect Disease, Guangzhou Women and Children’s Medical Center, Guangzhou Medical University, Guangdong Provincial Clinical Research Center for Child Health, No. 9 Jinsui Road, Zhujiang New Town, Tianhe District, 510623 Guangzhou, Guangdong China; 2 The First Affiliated Hospital of Jinan University, No. 613 West Huangpu Avenue, Tianhe District, 510630 Guangzhou, Guangdong China; 3 Guangzhou Medical University, No.1 Xinzao Road, Xinzao Town, Panyu District, 510182 Guangzhou, Guangdong China

## Abstract

**Background:**

Hirschsprung disease (HSCR) is a congenital intestinal malformation. Previous HSCR animal model needs invasive operation on adult animal. The aim of this study is to establish an early-onset animal model which is consistent with the clinical manifestation of HSCR patients.

**Methods:**

The neonatal mice were randomly divided into the benzalkonium chloride (BAC) group, treated with BAC via enema, and the control group, treated with saline. Weight changes, excretion time of carmine, CT scan, hematoxylin-eosin staining and immunofluorescence staining were used to evaluate the effect of the model. Differentially expressed genes (DEGs) in the HSCR mice were analyzed by using DAVID 6.8 database and compared with DEGs from HSCR patients.

**Results:**

The weight of mice was lower and the excretion time of carmine was longer in the BAC group. Moreover, distal colon stenosis and proximal colon enlargement appeared in the BAC group. Neurons in the distal colon decreased significantly after 4 weeks of BAC treatment and almost disappeared completely after 12 weeks. Transcriptome profiling of the mouse model and HSCR patients is similar in terms of altered gene expression.

**Conclusions:**

An economical and reliable HSCR animal model which has similar clinical characteristics to HSCR patients was successfully established.

**Impact:**

- The animal model of Hirschsprung disease was first established in BALB/c mice.
- This model is an animal model of early-onset HSCR that is easy to operate and consistent with clinical manifestations.
- Transcriptome profiling of the mouse model and HSCR patients is similar in terms of altered gene expression.

## Introduction

Hirschsprung disease (HSCR) is a congenital digestive system malformation which is mainly caused by abnormal migration and development of enteric neural crest cells (ENCCs) in the embryonic stage, resulting in the absence of ganglion cells of different lengths in the distal colon.^1–3^ The diseased bowel segment that lacks ganglia shows spastic stricture, causing abdominal distension and constipation.^4^ Clinically, HSCR patients usually develop vomiting, abdominal distension, constipation and other symptoms at the early stage of life and may be accompanied by serious complications, with a high mortality rate.^5^ The pathogenesis of HSCR remains unknown, and genetic and environmental factors are considered to be important influencing factors.^6,7^ Researchers are still committed to the study of the etiology, mechanism and treatment of HSCR.

Animal models are a critical tool for understanding the anatomy and pathophysiology of disease and exploring new treatments. Gene knockout mice are widely used in disease research, but the models based on single gene knockout have certain limitations in the study of complex polygenic diseases such as HSCR. Moreover, the single-gene knockout model is not suitable for the study of HSCR diseases caused by environmental factors. Besides, most knockout mice do not live long enough to meet the needs of experiments that focus on long-term outcomes.^8^ Therefore, a non-gene editing model with a good phenotype is in great demand. Since Sato et al.^9^ first reported that benzalkonium chloride (BAC) can cause the disappearance of intestinal ganglions in 1978, chemical induction animal models have received attention. As early as 2002, Yoneda et al.^10^ performed laparotomy using BAC on mice aged 12–16 weeks to establish an HSCR model. In 2014, Wagner et al.^11^ successfully established an animal model of HSCR in adult female Lewis rats by applying BAC to the descending colon through laparotomy. There are other models reported in adult mice with similar methods.^12,13^ So far, the chemical induction modeling approach is still dominated by invasive open surgery, and there is a lack of modeling in younger mice. Therefore, on the basis of previous research experience, we hope to explore an appropriate modeling method for neonatal mice to simulate the clinical characteristics of early-onset HSCR as much as possible and at the same time, to make an attempt in a simple and non-surgical way, so as to enrich the selection of HSCR animal models in future research.

In this study, an HSCR animal model with a simple method and early onset as a clinical manifestation of HSCR patients was established using neonatal BALB/c mice. During model establishment, BAC was applied to the colon via enema, which avoids the difficulty of surgical operation and reduces the mortality rate of animals. Besides, the selection of neonatal mice with immature intestinal nerve development fits the clinical feature of HSCR patients, which is beneficial to study the pathological mechanism of HSCR and evaluate new diagnosis and treatment methods.

## Materials and methods

### Experimental animals and protocols

Neonatal BALB/c mice at 5–8 days of age were selected [provided by Guangdong Yukang Biotechnology Co., Ltd., Production License: SCXK (Guangdong) 2020-0054] and randomly divided into the benzalkonium chloride (BAC) group (*n* = 12) and the control group (*n* = 12). All neonatal mice were breastfed by female adult mice, and adult mice were fed with mouse feed and water freely. All of them were kept in a specific pathogen-free environment (12 h light/12 h dark; temperature 25 ± 2 °C; humidity 55 ± 5%). All operations are in accordance with the regulations on the feeding and use of laboratory animals. This study was approved by the Animal Care and Use Committee of Guangzhou Women and Children Medical Center (NO. 2016042036).

### Modeling approach

#### Enema tool preparation

Type 1: Select a number of 2 cm polytetrafluoroethylene (PTFE) tubes with outer diameters of 0.3, 0.5 and 0.6 mm, and insert them in turn from thin to thick according to the outer diameter. Then connect the 0.6 mm outer diameter end with no.6 needle, as shown in Supplementary Fig. 1A, and then use a 1 mL syringe for an enema.

Type 2: Type 1 enema tool without connecting 0.3 mm PTFE tubes, as shown in Supplementary Fig. 1B.

#### BAC treatment method

The neonatal mice were fixed with tape and connected to a syringe containing BAC (Sigma-Aldrich, St. Louis, MO, Batch Number: 12060) with the enema tool; the anterior segment was lubricated with paraffin oil and was finally inserted into the anus of the mice [Supplementary Fig. 1C]. In the BAC group, BAC treatment was performed at the first week of life (5–8 days of age, 4–5 g of weight in neonatal mice) and repeated in the second week (12–15 days of age, 6–10 g of weight) and in the third week (19–21 days of age, 8–12 g of weight) to induce colon neurons damage. In the first week and the second week, the type 1 tool was used, and the depth of the anal implantation was 0.5–1 cm and 1–1.5 cm, respectively. Then, 20 μL of 0.2% BAC solution would be injected twice in the first week and three times 20–40 μL of 0.2% BAC solution in the second week with a 10-min injection interval. In the third week, a type 2 enema tool was selected to be inserted through the anus at a depth of 1.5–2 cm, and four times 40–60 μL of 0.5% BAC solution were given with an interval of 10 min. In the control group, 0.9% saline (Kelun Pharmaceutical, China, Batch Number: AB8189) was used to replace BAC, while the rest processes were the same as the treatment group [Table 1 and Supplementary Fig. 2].

**Table 1:** The content of model establishing method.

	Age in days (d)	Weight (g)	Enema tool	Insertion depth (cm)	BAC concentration	BAC dosage (μL)	Frequency
First enema	5–8	4–5	Type 1	0.5–1	0.2%	20	2
Second enema	12–15	6–10	Type 1	1–1.5	0.2%	20–40	3
Third enema	19–21	8–12	Type 2	1.5–2	0.5%	40–60	4

### Model identification

#### Body weight change recording

The body weights of each animal were measured to understand the health status of mice.

#### Carmine lavage and red feces excretion

Carmine lavage experiment was conducted in the BAC group and the control group to evaluate the intestinal function: 4 weeks and 12 weeks after the BAC treatment, two groups of mice were alone cage, 1 mL carmine (Sigma-Aldrich, St. Louis, MO, Batch Number: SHBL4031) solution (6 mg/L) was used to perform lavage. At last, the time used for red feces excretion was calculated and compared.

#### Colography and micro-CT scanning

Three-dimensional reconstruction of colography of mice after micro-CT scanning reflected the intestinal lesions of mice: 12 weeks after the BAC treatment, the mice were anesthetized by intraperitoneal injection of 10 μL/g tribromoethanol (Nanjing Aibei Bio-Technology Co., LTD., China, Batch Number: 2039A). Later, 300–500 μL of 30% meglumine diatrizoate (Hanfeng, China, Batch Number: H20034058) would be used to highlight the intestine via enema. The mice were placed into micro-CT after waiting 2 min for better distribution of meglumine diatrizoate in the intestine.

#### Phenotype and histological analyses

Intestinal tissue samples were collected at 4 weeks and 12 weeks after the BAC treatment for phenotype identification. The tissue samples would then be fixed, embedded and sectioned. Immunofluorescence (IF) staining of protein gene product 9.5 (PGP9.5) polyclonal antibody (Abcam, Cambridge, MA, Batch Number: AB8189) and hematoxylin-eosin (H&E) staining were performed for detailed histologic examination.

### Analysis and comparison of differentially expressed genes between the HSCR mouse model and human HSCR disease

#### RNA sequencing (RNA-seq)

Colon tissues of 12 weeks BAC-treated mice and control mice were extracted; similarly, ganglionic segment and aganglionic segment tissue samples from HSCR patients were also obtained. This study has been approved by the Ethics Committee of Guangzhou Women and Children’s Medical Center, and the written consent of the guardian of patients with HSCR has also been obtained. The total RNA of each sample was isolated and purified. RNA purity (OD260/280 and OD260/230 ratio) was detected by Nanodrop ND-1000 (Thermo Fisher Scientific, Waltham, MA), and RNA integrity was assessed by Agilent 4200 TapeStation (Agilent Technologies, Santa Clara, CA). LncRNA library was constructed by removing ribosomal RNA (rRNA). cDNA size in 200–300 bp was screened and purified PCR amplified library was obtained. The library was quantified using Qubit 2.0 Fluorometer (Thermo Fisher Scientific, Waltham, MA) and diluted to 1.5 ng/μL. The insert size was detected by Agilent 4200 TapeStation. The Illumina NovaSeq 6000 (Illumina, San Diego, CA) platform was used to generate PE150 NGS sequences.

#### RNA-seq bioinformatics

Raw FASTQ files were first quality controlled by removing joints, repeated sequences and low-quality sequences. Reference genomes and gene model annotation files (GRCh37 and GRCm38) were selected. HISAT2 (v2.1.0) was used for read mapping. The expression level of genes was obtained using the featureCounts software (v2.0.3). Differential genes among samples were analyzed using R package edgeR. A *P*-value less than 0.05 was treated as nominally significant. Finally, DAVID 6.8 database (https://david.ncifcrf.gov/) was used to conduct GO enrichment analysis.

#### Statistical analysis

Statistical analysis was performed using GraphPad Prism Software (v8.4.3). The numerical data were expressed as mean ± standard deviation (mean ± SD). Two-way ANOVA was used for the comparison of weight gain between the BAC treatment group and the control group, and Student’s *t*-test was used for the comparison of the remaining data between the two groups. A *P*-value less than 0.05 indicated that the difference was statistically significant.

## Results

### Survival analyses

A total of 24 neonatal mice were included in this study, and they were divided into the control group and the BAC group by random number method. In the control group, one mouse died after the first enema, and the other 11 mice survived. In the BAC group, one mouse died after the first enema, two mice died after the second enema, and nine mice survived.

### BAC treatment affected weight gain suggesting the model’s disease status

**Fig. 1:**
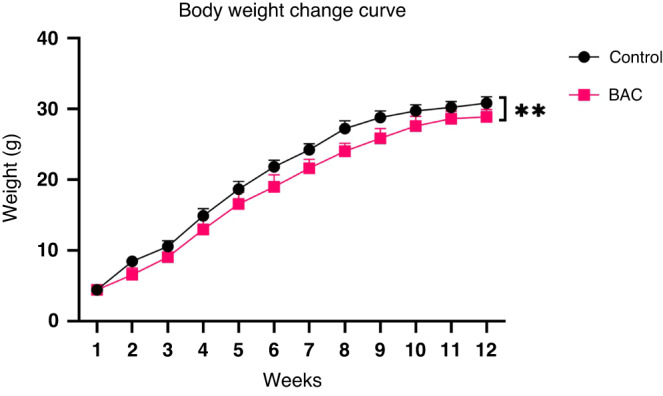
Body weight change of mice in the treatment group and control group. After 12 weeks, the weight of both control and BAC treated mice gradually increased, but the weight of BAC treated mice was significant lower than that of the control group.

The body weight of the BAC group mice increased slowly after the first BAC treatment, and the final body weight was significantly lower than that in the control group. The body weight change of mice is shown in Fig. 1.

### BAC treatment resulted in prolonged excretion time, indicating intestinal peristalsis dysfunction

**Fig. 2:**
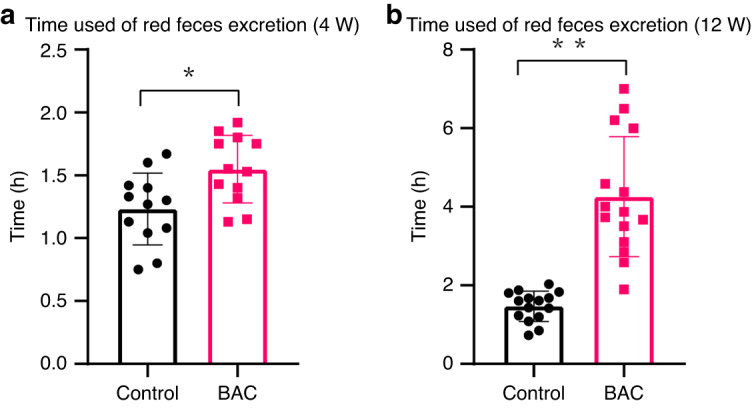
Carmine excretion time in the treatment group and control group. **a** Carmine excretion time collected at 4 weeks after the BAC treatment. **b** Carmine excretion time collected at 12 weeks after the BAC treatment.

Carmine gavage test was performed in mice of two groups at 4 weeks and 12 weeks after the end of BAC treatment. The excretion time of carmine in the two groups can be used to evaluate the intestinal peristalsis function, thus reflecting the ablation status of intestinal neurons. Compared with the control group, mice in the BAC group showed slightly prolonged excretion time at 4 weeks (1.23 ± 0.29 h vs. 1.55 ± 0.27 h, *P* < 0.05), which indicated intestinal peristalsis dysfunction [Fig. 2a]; At 12 weeks, the intestinal peristalsis function of mice in the BAC group was severely impaired (1.47 ± 0.38 h vs. 4.26 ± 1.53 h, *P* < 0.01) [Fig. 2b].

### Colography and micro-CT scan after BAC treatment showed megacolon formation

**Fig. 3:**
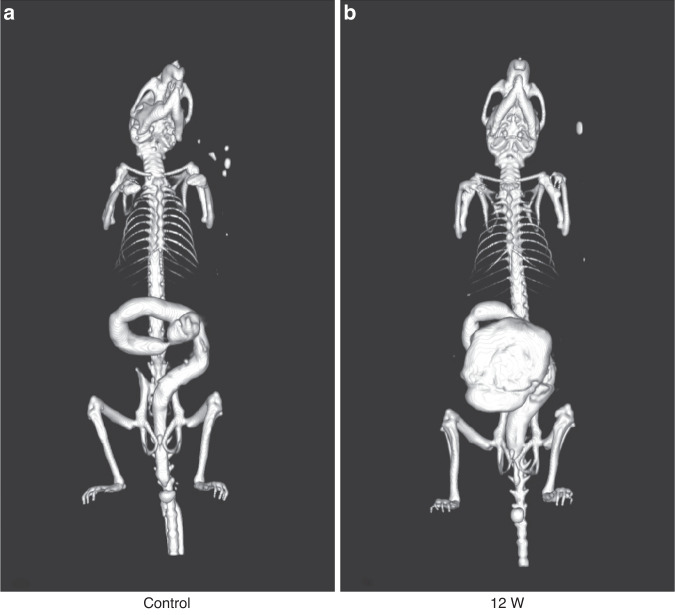
Three-dimensional reconstruction of micro-CT scanning image in treatment group and control group at 12 weeks after BAC treatment. **a** The distal colon of the control group was uniform in size. **b** The contrast agent gradually decreases or even disappears at the end of the distal colon of the treatment group but increases in the proximal colon.

Three-dimensional reconstruction of colography of mice in this study was conducted after micro-CT scanning. Stenosis occurred in the distal colon of the BAC group, and a large amount of contrast agent accumulated in the intestinal lumen of the proximal colon, clearly indicating the local colon enlargement and irregular deformation [Fig. 3a], while the distal colon of the control group was uniform in thickness, and intestine enlargement was not observed [Fig. 3b].

### Significant phenotype of megacolon was observed after 12 weeks of BAC treatment

**Fig. 4:**
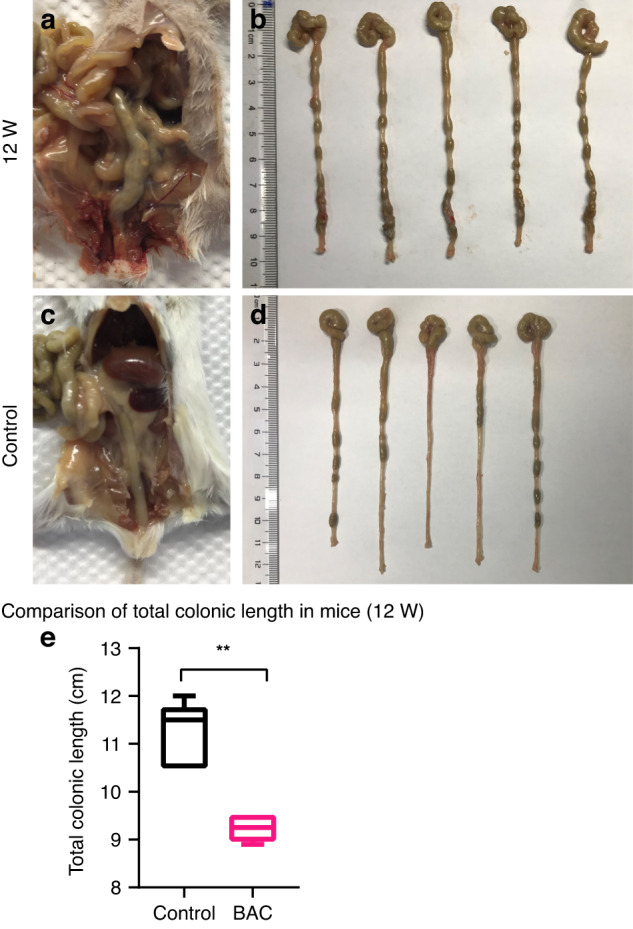
Gross morphological observation of colon tissue in the treatment group and control group at 12 weeks after BAC treatment. **a**, **b** Fecal accumulation was obvious in the distal colon of the treatment group, forming an expanded colon with irregular shape and uneven thickness. **c**, **d** The size of the distal colon of the control group was uniform and smooth, without obvious fecal accumulation. **e** The length of the colon was found to be shorter in the treatment group than in the control group.

The morphology of the colon in the 12th week after BAC treatment was observed. In the BAC group, fecal accumulation was obvious in the distal colon and the colon was obviously divided into dilated segment and narrow segment, forming an expanded colon with irregular shape and uneven thickness [Fig. 4a, b]. In contrast, the distal colon in the control group was uniform and smooth [Fig. 4c, d]. Besides, the length of the colon was found to be shorter in the BAC group than that in the control group (9.28 ± 0.25 cm vs. 11.20 ± 0.67 cm, *P* < 0.01) [Fig. 4e].

### Histological analyses have proved the success of the HSCR modeling

**Fig. 5:**
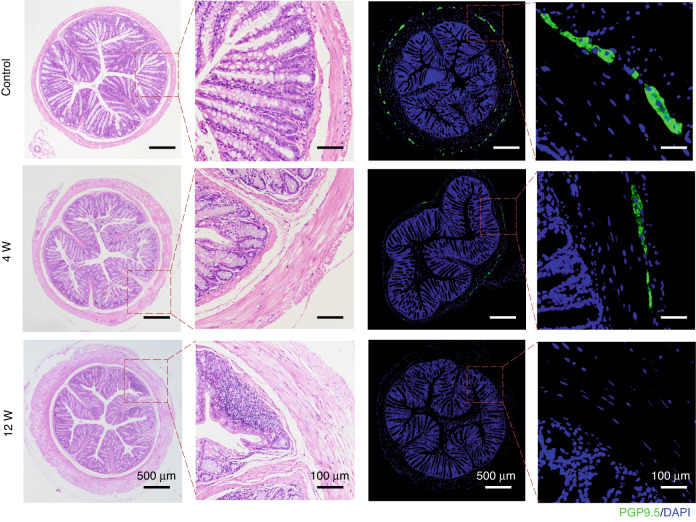
H&E and IF staining of colon tissue in the control group and narrow segment tissue in the treatment group. H&E staining showed that the ganglion of the intermuscular plexus was ablated, but the other tissues were intact. In addition, thickening of muscle layer tissue and a small amount of inflammatory cell infiltration were found (objective lens: ×10). IF staining showed that the expression of ganglion-specific marker PGP9.5 gradually decreased to disappearance (objective lens: ×10).

HE and IF staining of colon tissue in the control group and the BAC group (4 weeks and 12 weeks after BAC treatment) were performed. Compared with the control group, the basic morphological structure of the mucosal layer, submucosal layer, musculature layer and serosal layer of intestinal tissue at 4 weeks and 12 weeks after BAC treatment were basically normal, but inflammatory cell infiltration was observed in the BAC group. IF staining of neurons with intestinal neuron-specific marker PGP9.5 showed that intestinal neurons in the control group were evenly distributed and abundant, and colon neurons in cross section were stained into perfect circular rings. However, the expression of PGP9.5 decreased significantly, and only a few neurons were scattered and unevenly distributed in the BAC group. Neuronal expression was undetectable in intestinal tissue in the BAC group after 12 weeks of BAC treatment, indicating the success of this approach in inducing an animal model of HSCR [Fig. 5].

### Analysis and comparison of differentially expressed genes suggested the similarity between the HSCR mice and the HSCR patients

**Fig. 6:**
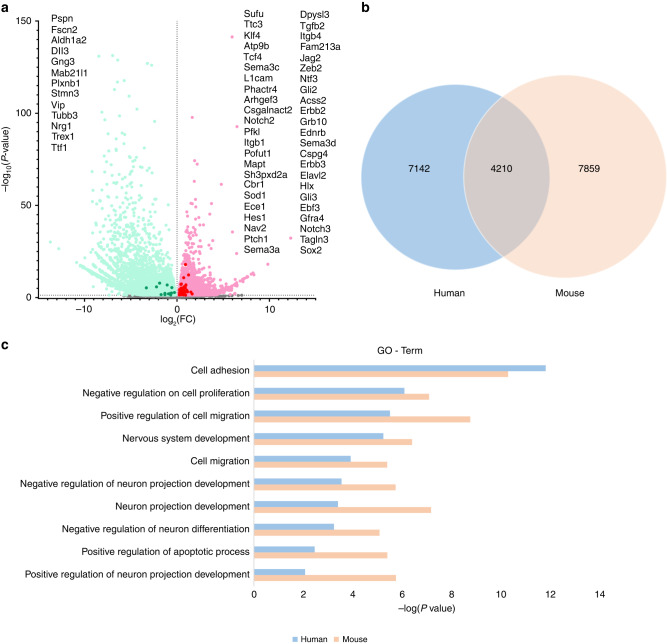
Differentially expressed genes (DEGs) in BAC-treated and control mice, and comparison with DEGs in dilated and narrowed segments in HSCR patients. **a** Volcano plot of DEGs from BAC-treated and control mice. Dots in green indicates the genes with low expression, and red indicates the genes with high expression. The dots marked with dark colors represent the genes associated with HSCR, which are listed on either side of the volcano plot. **b** Venn diagram of overlapped DEGs in HSCR patients (Blue) and mouse model (Orange). **c** GO Term analysis on biological processes of overlapped DEGs in HSCR patients and mouse models.

In this study, the colon tissues of three BAC mice and four control mice were collected for RNA sequencing. A total of 12,069 DEGs were detected by differential expression analysis, including many currently recognized HSCR-related genes [Fig. 6a]. In addition, a total of 10 patients with HSCR were recruited. The ganglionic segment and aganglionic segment tissue were collected for RNA sequencing, and 11,352 DEGs were found by differential expression analysis (Data unpublished). After comparison of DEGs between the two sets of data, we found 4210 overlapping DEGs [Fig. 6b]. GO enrichment analysis showed that DEGs in HSCR mice and HSCR patients were mainly enriched in items that were closely related to cell adhesion, cell proliferation and migration, nervous system development, and neuronal projection [Fig. 6c]. The results indicate that the transcriptome profiling of the mouse model and HSCR patients in this study reveals similar alterations in gene expression.

## Discussion

HSCR is currently considered to be a developmental disorder of the enteric nervous system (ENS) due to disruption of ENCCs migration, proliferation, differentiation, survival, and/or apoptosis, resulting in a congenital lack of neurons in the intestinal tract.^3,14,15^ It is generally accepted that the ideal experimental model for studying human disease should simulate the disease in terms of anatomy, physiology and course. In this study, we established an HSCR animal model with simple operation, good phenotype, which replicates the etiology and the clinical symptoms of the human HSCR, including neurons in the distal colon decreased, poor weight growth, intestinal peristalsis dysfunction, distal colon stenosis and proximal colon enlargement, even biological information background.

Compared with gene knockout animal models, drug-induced animal models have the advantages of shorter modeling time and more economic benefits. BAC is a cationic surfactant that attaches to the cell membrane and causes its irreversible depolarization, which is more obvious on the cell membrane with a higher negative charge.^10^ Therefore, neurons in the intestine can be selectively destroyed.^12,16,17^ Sato et al.^9^ found that the application of BAC in the serosal layer of the colon also caused degeneration of other cells in the intestinal wall, but these lesions disappeared within a few weeks and the remaining tissues recovered completely except the intestinal wall neurons. So far, the mechanisms for the specificity of the neuron lesions after BAC exposure remain not well known. Sakata et al.^18^ suggested that this phenomenon might be related to the high sensitivity of intestinal neurons to BAC and the low resilience of neurons. Compared with the control group, except for the selective elimination of intestinal neurons, the basic morphological structures of the mucosal layer, submucosal layer, muscle layer and serosal layer in the BAC group were not damaged [Fig. 5]. To our knowledge, adult rats or mice are mainly selected in the current research on establishing the HSCR animal model by open surgery using BAC.^10–12,19,20^ However, adult rats or mice are not consistent with the clinical characteristics of HSCR patients, which manifests shortly after birth. In addition, surgical invasive procedures may lead to postoperative infection and intestinal adhesion, creating additional side effects and affecting phenotypic judgment. Besides, it has been reported that neurons can be detected again after nerve ablation in adult rats and mice using BAC.^21–23^ Studies have reported that nerve fibers regenerate and redominate muscles after BAC treatment. The regenerated neurons may come from peripheral neurons, submucosal nerve plexus or may be the result of migration and differentiation of neural progenitor cells in the transition region.^22^ However, after a single BAC treatment, the number of neurons regenerated in the previously aganglionic segment was much lower than in the control group.^22^ Theoretically, we selected neonatal mice with immature intestinal nerves^24,25^ for neuron ablation and repeated BAC treatment during modeling so that the intestinal neurons can be removed as far as possible.

In our modeling method, an enema of BAC solution was applied to reduce the impact of surgical treatment and improve the survival rate of the animals. In this study, it was found that, compared with the control group, the weight growth of mice in the BAC group was lower, which was consistent with the clinical symptoms of children with HSCR [Fig. 1]. Clinically, the weight growth of children with HSCR lags behind that of healthy children, and malnutrition is a common phenomenon in children with HSCR.^22^ Intestinal dyskinesia occurred in children with HSCR due to the loss of innervation of the colon and was later found to occur in BAC mice.^26^ The excretion time of carmine that evaluated the damage of intestinal peristalsis in mice was longer in the BAC group. Under our dynamic monitoring, with the progress of time, the excretion time in the BAC group became longer. This indicated that the damage to intestinal peristalsis function was gradually aggravated [Fig. 2]. In addition, the HCSR mice in our study had the typical intestinal phenotype consistent with children with HSCR, such as stenosis of the distal colon and swelling of the proximal colon with fecal accumulation.^2^ Immunofluorescence staining showed that colonic neurons were destroyed and disappeared in the BAC group [Fig. 5]. Therefore, we confirmed that the method successfully established the animal model of HSCR. Further, RNA-seq analysis of colonic tissues of the BAC group mice and HSCR patients showed that 4210 genes were co-enriched, and there were abundant co-enriched pathways [Fig. 6]. These findings indicate that the animal model established using BAC exhibits similar gene expression alterations to those observed in HSCR patients.

Taken together, we successfully established an experimental animal model of HSCR using BALB/c neonatal mice, enriching the existing animal models of HSCR and providing a reliable HSCR animal model for further research on its pathogenesis, treatment, clinical drug experiment, and so on.

### Supplementary Information

Supplement figures

## Data Availability

Data included in this manuscript are available upon request by contacting the corresponding author and will be freely available to any researcher wishing to use them for non-commercial purposes without breaching participant confidentiality.
