# Supplementary material for: Establishment and identification of an animal model of Hirschsprung disease in suckling mice
Source: Pediatr Res. 2023 Jul 17;94(6):1935–41. doi: 10.1038/s41390-023-02728-6 (PMC10665188; doi:10.1038/s41390-023-02728-6)
Supplement: Supplementary file 1 — Supplement figures [file 41390_2023_2728_MOESM1_ESM.pdf]

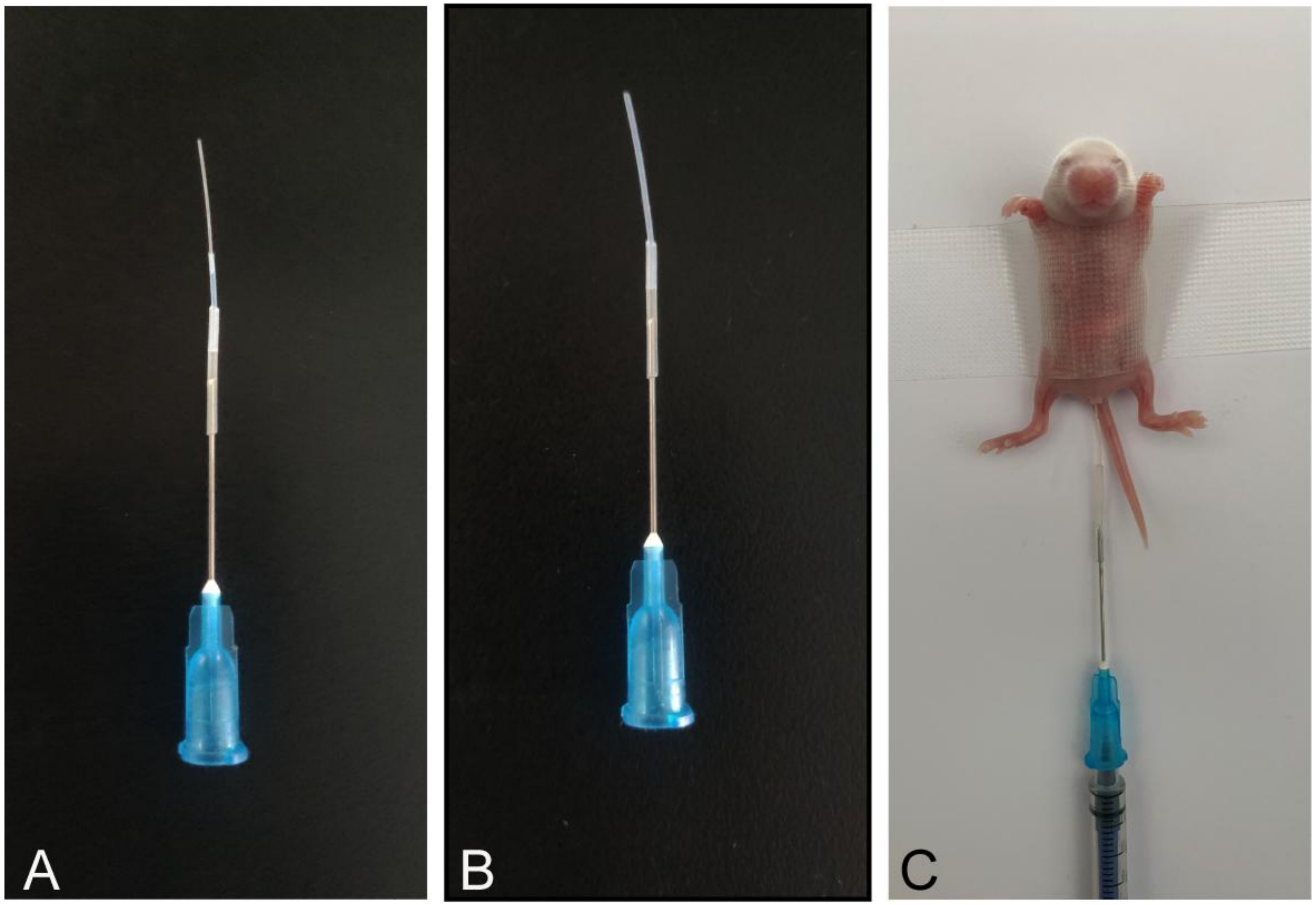

Supplement figure 1: Enema tool and BAC treatment diagram.

(A) Type 1 enema tool and (B) Type 2 enema tool. (C) BAC treatment operation.

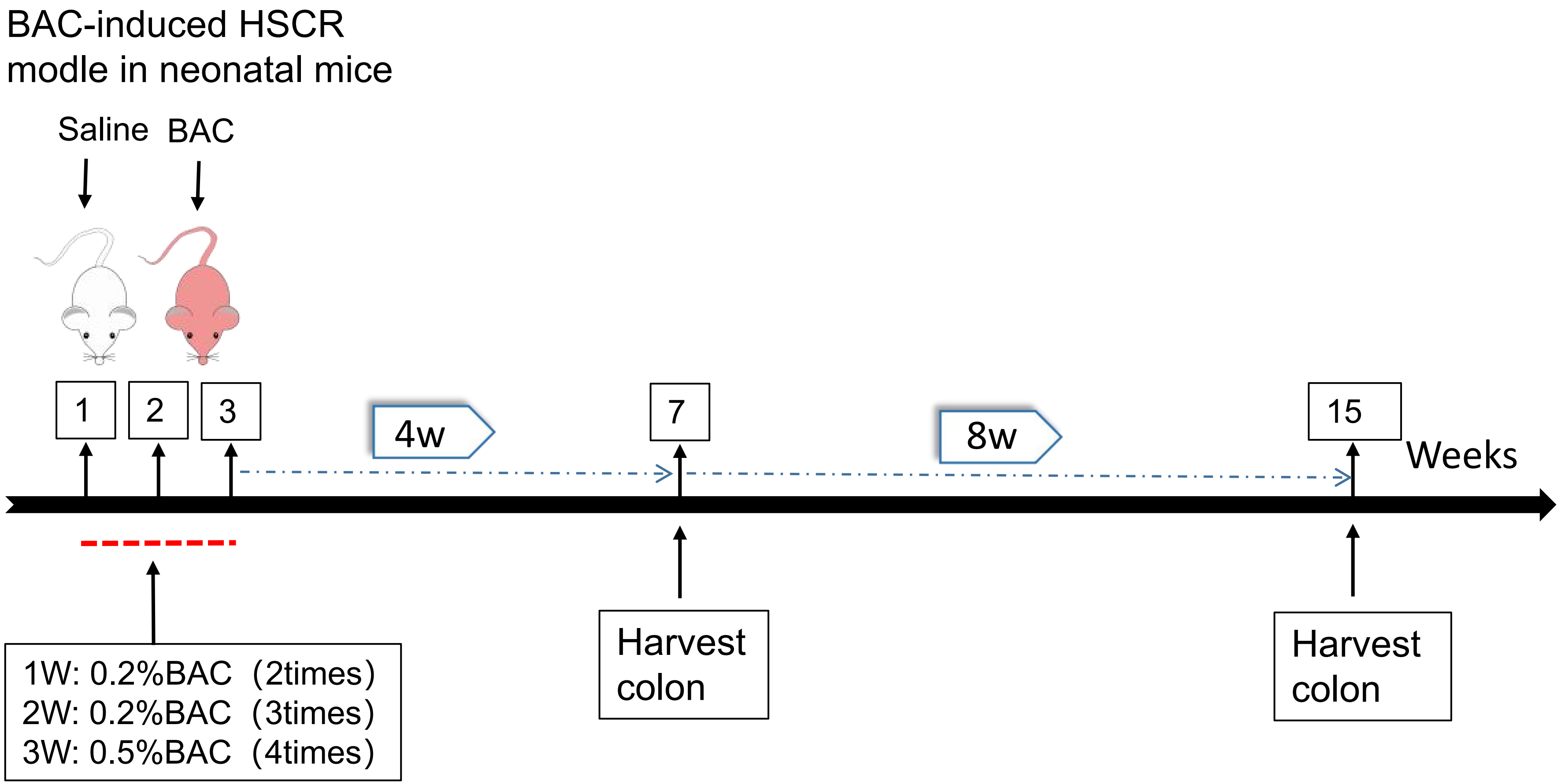

Supplement figure 2: Schematic diagram of building HSCR model in neonatal mice.
